# The impact of using an upper-limb prosthesis on the perception of real and illusory weight differences

**DOI:** 10.3758/s13423-017-1425-2

**Published:** 2018-01-19

**Authors:** Gavin Buckingham, Johnny Parr, Greg Wood, Samuel Vine, Pan Dimitriou, Sarah Day

**Affiliations:** 1 Department of Sport and Health Sciences, University of Exeter, Richard’s Building, St. Luke’s Campus, Exeter, UK; 2 Department of Health Sciences, Liverpool Hope University, Liverpool, UK; 3 Centre for Health, Exercise and Active Living, Manchester Metropolitan University, Crewe, UK; 4 Psychology Department, Heriot-Watt University, Edinburgh, UK; 5 National Centre for Prosthetics and Orthotics, Department of Biomedical Engineering, University of Strathclyde, Glasgow, UK

**Keywords:** Size–weight illusion, Object lifting, Amputees, Body representation

## Abstract

Little is known about how human perception is affected using an upper-limb prosthesis. To shed light on this topic, we investigated how using an upper-limb prosthesis affects individuals’ experience of object weight. First, we examined how a group of upper-limb amputee prosthetic users experienced real mass differences and illusory weight differences in the context of the ‘size–weight’ illusion. Surprisingly, the upper-limb prosthetic users reported a markedly smaller illusion than controls, despite equivalent perceptions of a real mass difference. Next, we replicated this dissociation between real and illusory weight perception in a group of nonamputees who lifted the stimuli with an upper-limb myoelectric prosthetic simulator, again noting that the prosthetic users experienced illusory, but not real, weight differences as being weaker than controls. These findings not only validate the use of a prosthetic simulator as an effective tool for investigating perception and action but also highlight a surprising dissociation between the perception of real and illusory weight differences.

The human hand provides the key way for humans to perform a range of dextrous skills, interact with the world, and perceive many properties of objects. When an individual loses a hand, a prosthetic device is often employed to replace the lost segment. Yet despite the apparent cosmetic and functional advantages of using a prosthesis after the loss of a limb, abandonment levels are surprisingly high (Biddiss & Chau, 2007). This may be, in part, because using an upper-limb prosthesis still leaves an amputee with impaired dexterity compared to preinjury levels (Cordella et al., 2016). Almost no research, however, has examined whether there are adverse perceptual consequences of using a prosthetic hand and arm. Here, we describe two experiments aimed at shedding light on how the use of a prosthetic device affects a critical perceptual process—the experience of an object’s weight.

Most research on prosthetic use examines prosthetic users functional ability to undertake activities of daily living (Haverkate, Smit, & Plettenburg, 2016; Wallace et al., 1999). However, our hands not only are tools to manipulate our environment but they also provide the main method to experience nonvisible properties of objects such as centre of mass, temperature, and weight (Gallace & Spence, 2014). Despite the critical role that our hands play in this regard, almost no work has examined how the use of an upper-limb prosthesis might affect the hedonic perception of manually acquired properties such as object weight. To date, the one study to examine perception in upper-limb prosthetic users has done so in the context of the perceptual sensitivity of weight discrimination. Wallace et al. (2002) compared the difference threshold of a single long-term prosthetic user to that of a group of controls using either their anatomical hand or a prosthesis simulator. The authors found that the long-term prosthetic user was just as proficient as an intact control group at discriminating between objects of different mass. However, when using the prosthetic simulator, controls were significantly worse compared with when they used their anatomical hand. Despite these promising results, examining perceptual sensitivity (i.e., difference limen) is distinct examining a hedonic perceptual experience. Felt heaviness is a unique perceptual property, with the experience of how heavy an object feels reflecting a combination of bottom-up and top-down processes such as the lifter’s fatigue (Burgess & Jones, 1997), their grip aperture (Koseleff, 1957), the friction coefficient of the object (Flanagan & Bandomir, 2000), and the force used to lift the object (van Polanen & Davare, 2015). One compelling example of the subjective nature of weight perception comes from the size–weight illusion (SWI), where small objects feel heavier than equally weighted larger objects (Charpentier, 1891; Flournoy, 1894). Explanations for this illusion fall broadly into two categories. Bottom-up theories propose that individuals detect some property instead of an object’s mass, such as density (Ross & Di Lollo, 1970), inertia (Amazeen & Turvey, 1996), or throwability (Zhu & Bingham, 2011), when they attempt to judge its weight. Top-down explanations, by contrast, suggest that the illusion is a by-product of how sensory information is combined with prior expectations (Buckingham, 2014). In this context, the small objects feel heavier than equally weighted larger objects because the lifter expected them to be lighter and subsequently experience a contrast with their prior expectations. The role of prior expectations causing at least a portion of the experience of the SWI, and related effects with material cues, is well-established (Buckingham & Goodale, 2010; Buckingham, Ranger, & Goodale, 2011; Flanagan, Bittner, & Johansson, 2008). Exactly what constitutes a prior expectation in this context is, however, far more contentious (Buckingham, 2014; Buckingham & MacDonald, 2016; Dijker, 2014; Peters, Ma, & Shams, 2016; Vicovaro & Burigana, 2016).

There is emerging evidence that an individual’s perception of how heavy an object feels is linked to their body schema. Case, Wilson, and Ramachandran (2012) examined the SWI in individuals with anorexia nervosa—a psychiatric disorder characterised by a distorted body image—noting that individuals with anorexia experienced a substantially smaller SWI than healthy controls experienced. While the authors attributed this the reduced SWI in individuals with anorexia nervosa to an impairment in visuo-proprioceptive integration, it is also feasible that this reduced illusion reflected an impairment in the updating of body representation in this population. This interpretation is supported by several perceptual studies explicitly manipulating the apparent size of the lifting hand. Building on the ecologically inspired concept of the body as a perceptual reference point for perceptual judgements (Linkenauger, Leyrer, Bülthoff, & Mohler, 2013; Linkenauger, Ramenzoni, & Proffitt, 2010), recent work has shown that the SWI can be induced by rescaling the apparent size of the lifting hand. For example, magnifying the hand to make objects appear relatively smaller makes objects feel reliably heavier (Linkenauger, Mohler, & Proffitt, 2011). The most stark demonstration of how weight perception can be driven by changes in body representation comes in the context of the ‘rubber-hand illusion’, where noncorporeal plastic or rubber hands are incorporated into the body schema, feeling like an anatomical hand. Using this technique, Haggard and Jundi (2009) showed that experience weight was related to the size of the new effector, such that incorporating a large hand into one’s body schema resulted in objects feeling heavier than when incorporating a small hand.

It is well established that humans have flexible mechanisms for updating how they represent their bodies (Azañón et al., 2016). This plasticity allows us to incorporate tools into our body schemas (Maravita & Iriki, 2004; Martel, Cardinali, Roy, & Farnè, 2016) and adapt to dramatic changes in our physical bodies, such as the loss of a limb (Canzoneri, Marzolla, Amoresano, Verni, & Serino, 2013). The use of prosthetic limbs offer a unique insight into the plasticity of body representation, as they bridge the conceptual gap between a limb and a tool (van den Heiligenberg, Yeung, Brugger, Culham, & Makin, 2017). Here, we present the first experiments to examine how the use of an upper-limb prosthesis affects the experience of an object’s weight in the context of real and illusory weight differences. First, a group of amputee prosthetic-hand users and a group of nonamputee controls lifted and reported the felt weight of a range of objects which independently varied in mass and volume (and should thus induce the SWI). Next, we examined real and illusory weight perception in a group of able-bodied individuals who lifted our test objects with a myoelectric prosthetic simulator, again comparing their experience of object weight to a separate group of control subjects lifting with their anatomical hand. We predicted that experienced prosthetic users would experience real and illusory weight differences in a fashion similar to control groups, reflecting the impact that their unique level of expertise and experience with their prosthesis would affect the embodiment of their prosthetic hand. Further, if ‘normal-seeming’ hedonic perception is a natural by-product of long-term experience with their device, we would expect that the users of a prosthetic simulator to experience real weight differences and illusory weight differences less vividly than individuals using their anatomical hand.

## Experiment 1: Upper-limb amputees using prosthetic hands

### Materials and methods

#### Participants

In this experiment, we recorded the perceptual experience of object heaviness in nine upper-limb amputees lifting with their prosthetic hand (upper-limb amputee group) and 20 intact participants lifting with their preferred hand (control group).

The upper-limb amputee group comprised seven males and two females, aged between 20 and 75 years (mean = 63.3 years, *SD* = 15.2). They were recruited from various regions of Scotland, with the inclusion criteria of an upper-limb absence for >10 years, and possessing a prosthetic device which they were comfortable wearing and were confident using to lift objects. All participants were traumatic amputees and wore prosthetic devices to replace their dominant hand. Further information of the range of prosthetic devices can be found in Table 1.

**Table 1:** Prosthesis information about the nine upper-limb amputees recruited in this study

Site of amputation	Socket suspension	Terminal device
Trans-radial	Liner and pin	Cosmetic foam hand
Trans-radial	Liner and pin	Cosmetic foam hand
Trans-radial	Skeletal self-suspending	I-limb ultra myoelectric hand
Trans-radial	Liner and pin	Standard myoelectric hand
Trans-radial	Harness	Voluntary-opening hand
Trans-radial	Skeletal self-suspending	Cosmetic foam hand
Trans-humeral	Harness	Voluntary-opening hand
Trans-humeral	Harness	Cosmetic foam hand
Trans-humeral	Harness	Voluntary-opening hand

The control group comprised 11 males and nine females, aged between 20 and 59 years (mean = 43.1 years, *SD* = 17.4), and were recruited from staff and student populations at the University of Strathclyde. All participants gave informed consent prior to testing, and all procedures were approved by the local research ethics boards at the University of Strathclyde.

#### Materials

**Fig. 1:**
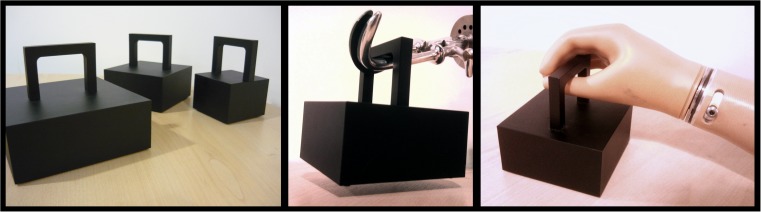
One set of the small, medium, and large objects used in Experiment 1, as well as examples of how the objects were lifted with different types of prostheses

Participants lifted and judged the weight of nine custom-manufactured cuboids made from dark grey polyvinylchloride (see Fig. 1), which varied independently in volume (three small, three medium, and three large) and mass (three light, three middle weight, and three heavy). The light objects were 500 g, the middle-weight objects were 600 g, and the heavy objects were 700 g. The small objects were 7 cm × 7 cm × 5 cm, the medium objects were 10 cm × 10 cm × 5 cm, and the large objects were 13 cm × 13 cm × 5 cm. All the objects had the same-sized handle attached to the top surface designed to accommodate a range of prosthesis terminal devices.

#### Procedure

The task required participants to lift and judge the weight of nine cuboids multiple times. On each trial, participants were asked to close their eyes whilst one of the objects was placed in front of them on a table surface. They were then asked to open their eyes and lift the object using the handle. No constraints were placed upon the style, height, or time course of the lift—participants were encouraged to lift in a natural fashion as they would outside of the laboratory. In practice, the style of lift used by participants in the amputee group tended to reflect the characteristics of their prosthesis terminal device (for some examples, see the middle and right panels of Fig. 1).

Once they had reached the apex of their lift, participants were asked to give an arbitrary magnitude estimation (Zwislocki & Goodman, 1980) of the object’s weight by providing an unconstrained numerical rating which represented how heavy the object felt, with larger numbers representing heavier-feeling objects. They were informed that there was no upper or lower limit to this scale and that they could use fractions if they felt it appropriate. Once they had reported the felt heaviness of the object, they lowered it to the table surface and closed their eyes in preparation for the next trial. This procedure was undertaken four times for each object in a random order, for a total of 36 lifts in a single session lasting 20–45 minutes.

Prior to statistical analysis, the perceptual ratings were normalised *Z* distribution in order to account for individual differences in participants’ rating scales. All statistical analyses were undertaken using JAMOVI Version 0.7.0.2. Significant main effects were followed up with post hoc *t* tests, and null findings of particular interest were followed up with Two One-Sided Test procedures to establish equivalence (Lakens, 2017) . An alpha of .05 was used to indicate significance in all inferential tests. In tests where sphericity was violated, the Greenhouse–Geisser correction was used (data from this experiment can be found at https://osf.io/yzv5a/).

### Results

**Fig. 2:**
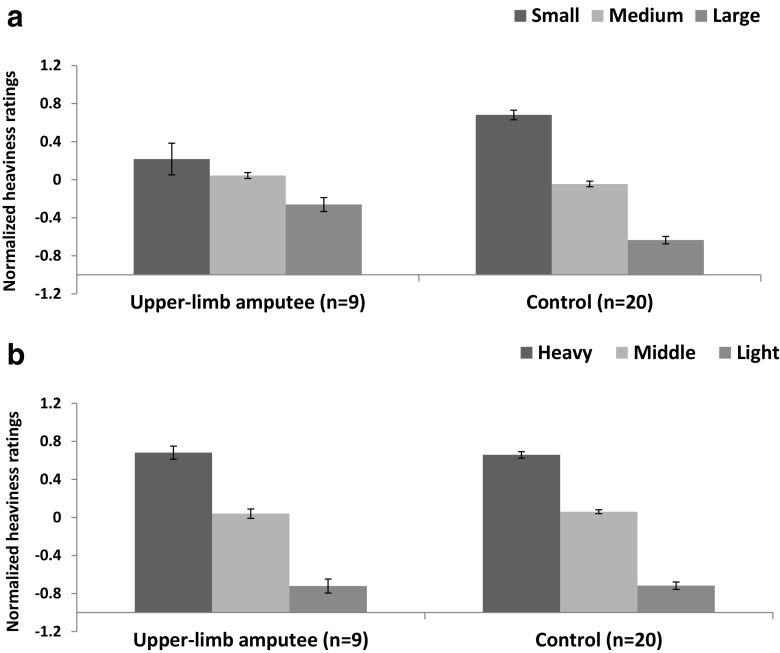
Heaviness ratings, normalized to a Z distribution within subject, as a function of (**a**) relative object size and (**b**) relative object weight. Error bars show standard error of the mean

We first examined the normalised heaviness ratings in a 3 (size) × 3 (weight) × 2 (group) mixed-factorial ANOVA. There were significant main effects of size, *F*(1.2, 32.6) = 43.1, *p* < .001, ω^2^ = .52 (see Fig. 2a) and weight, *F*(1.6, 41.8) = 313.3, *p* < .001, ω^2^ = .92 (see Fig. 2b), and no interaction between size and weight (*p* = .44). Although there was no main effect of group (*p* > .99), we found a significant Size × Group interaction, F(1.2 ,32.6) = 9.7, *p* < .005, ω^2^ = .11, suggesting that there were differences in the magnitude of the SWI experienced by our different groups.^1^ By contrast, there was no significant Weight × Group interaction (*p* = .88), suggesting that there was no difference in how the groups experienced real mass differences. Furthermore, there was no significant three-way Size × Weight × Group interaction observed in this study (*p* = .35).

**Fig. 3:**
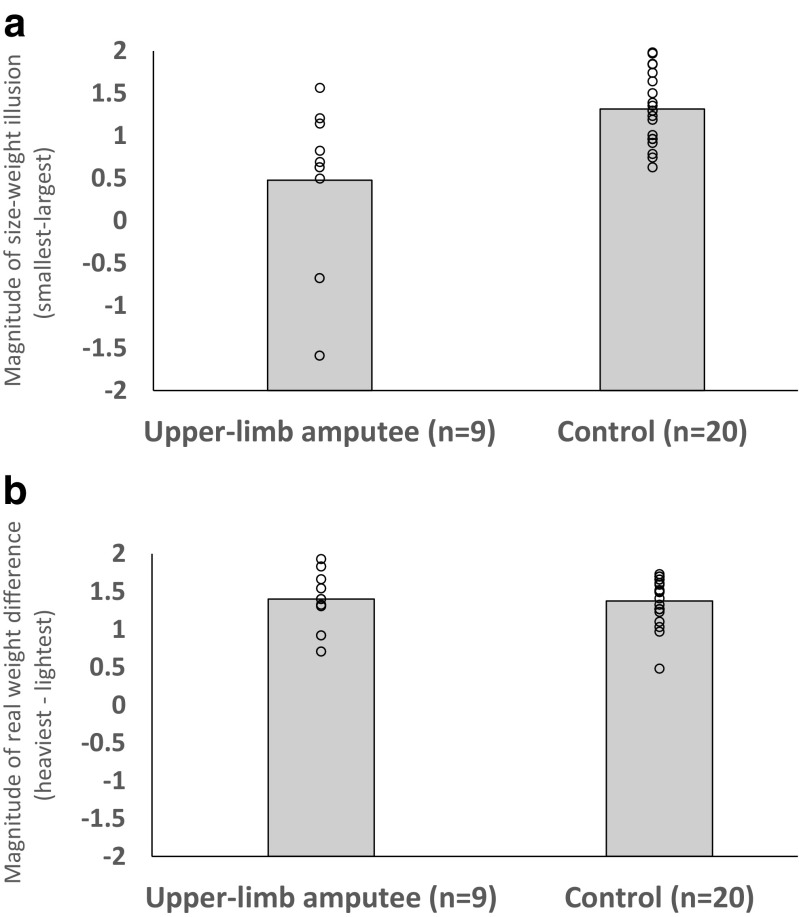
**a** Magnitude of the size–weight illusion experienced by both groups (calculated by subtracting the average ratings given to the largest objects from the average ratings given to the smallest objects). **b** Magnitude of the real weight difference experienced by both groups (calculated by subtracting the average rating given to the lightest objects from the average rating given to the heaviest objects). Positive numbers indicate an effect in the expected direction (i.e., experiencing heavy objects as feeling heavier than lighter object and experiencing smaller objects as feeling heavier than larger objects). The circles show individuals’ difference scores for these metrics

Next, we calculated difference scores to better visualise how the magnitude of the perceptions of (1) the SWI and (2) real weight differences varied between our groups. We defined the magnitude of the SWI experienced by each individual as the average rating given to all the large objects subtracted from the average rating given to all the small objects. Next, we examined how dramatic the real weight difference experienced by each individual was by subtracting the average rating given to the lightest objects from the average rating given to the heaviest objects. Independent-samples *t* tests confirmed that the upper-limb amputee group experienced a smaller SWI than controls, *t*(27) = 3.2, *p* = .003, *d* = 1.29 (see Fig. 3a), but showed no difference between how these groups experienced a real weight difference, *t*(27) = 0.20, *p* = .85, *d* = 0.08 (see Fig. 3b). To clarify this null finding, we then examined the real weight difference comparison with the Two One-Sided Test procedure based on Welch’s *t* test (Lakens, 2017; Walker & Nowacki, 2011). This test indicated that the observed effect size for this comparison (*d* = 0.08) was significantly within the equivalence bounds of *d*: −1.29 and 1.29, *t*(12.6) = 2.97, *p* = .007, suggesting that both groups had a similar experience of a real mass difference.

Finally, we compared the real and illusory weight perception metrics in a 2 (metric) × 2 (group) mixed-factorial ANOVA, noting that there was a significant interaction between metric and group, *F*(1, 27) = 11.9, *p* < .005, ω^2^ = .15. This final analysis confirms the presence of a dissociation between real and illusory weight differences experienced by our groups.

## Experiment 2: Nonamputees using an upper-limb prosthetic simulator

Experiment 1 demonstrated that using a prosthetic hand led to a reduction in the magnitude of the perceptual SWI compared to a control group, with no concomitant reduction in the experience of real weight differences. To clarify these findings, we next measured perceptions of heaviness in a larger group of intact individuals lifting illusion-inducing objects with a myoelectric prosthetic simulator worn over their anatomical hand.

### Materials and method

#### Participants

Here, we compared the perceptual experience of a group of nonamputee individuals using a myoelectric prosthetic hand to that of a new control group. The prosthetic simulator group comprised 11 males and nine females, aged between 21 and 37 years (mean = 27 years, *SD* = 4.4). The control group comprised nine males and 11 females, aged between 18 and 37 years (mean = 21.6 years, *SD* = 4.6). All participants in both groups considered themselves to be right-handed. All participants were recruited from staff and student populations at Manchester Metropolitan University and Liverpool Hope University. All participants gave informed consent prior to testing, and all procedures were approved by the local research ethics boards at Manchester Metropolitan University and Liverpool Hope University.

#### Materials

**Fig. 4:**
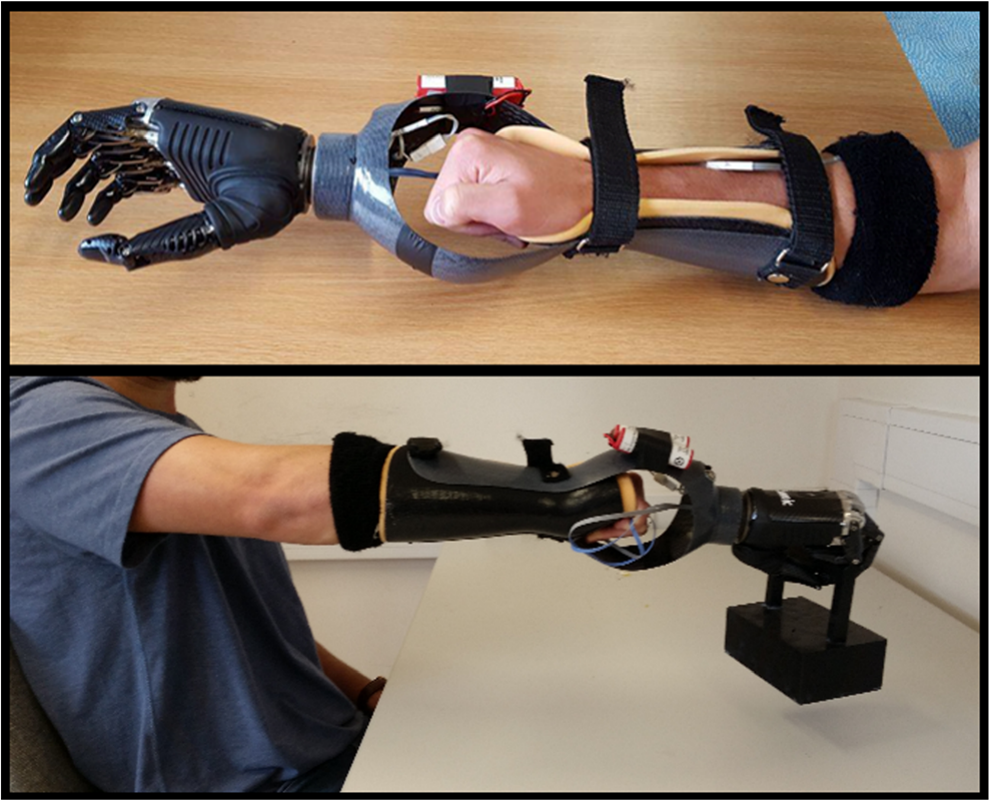
Bebionic myoelectric prosthetic simulator worn by individuals in Experiment 2

Participants in the simulator group wore a Bebionic™ (Steeper) myoelectric prosthetic hand simulator (see Fig. 4). In order to fit over the anatomical arm of able-bodied participants, the robotic hand was attached to the end of a carbon fibre trough, in which participants’ forearm and anatomical fist was positioned and fastened with Velcro straps (see Fig. 4). In total, this apparatus weighed just over 1 kg (1008.5 g). The grasping action was controlled by muscular contractions detected by two electrodes placed on the extensor and flexor muscles of the forearm, which were secured in place using a wristband. Activation of the extensors triggered the opening of the hand, whereas activation of the flexors triggered the closing of the hand. The grip pattern of the hand was set to the tripod grip.

In this second experiment, participants once again lifted and judged the weight of nine cuboids which varied independently in volume and mass. Due to fabrication constraints, these objects slightly differed in mass and volume to those in Experiment 1; the light objects were 400 g, the middle-weight objects were 500 g, and the heavy objects were 600 g; the small objects were 11.2 cm × 7.5 cm × 5.3 cm, the medium objects were 13.2 cm × 9.4 cm × 5.3 cm, and the large objects were 15 cm × 11.3 cm × 5.3 cm. All of the objects had an identical-sized handle mounted on centre of the top surface.

#### Procedure

Each participant was fitted with the simulator over their dominant hand and given time to learn how to manipulate the grasping action. They learned in an unsupervised way, over a period of 2–5 minutes, practicing the grasping action with minimal explicit instruction from the experimenters. To ensure that they had an adequate level of control to begin the experiment, the simulator users were asked to perform three consecutive sequences of opening and closing the hand. Each participant then had one practice trial of lifting the middle-weight and middle-sized object before the experiment began. Following this, the procedure for this experiment was identical to that of Experiment 1 (data from this experiment can also be found at https://osf.io/yzv5a/).

## Results

One participant was removed from the prosthetic simulator group for reporting an average SWI greater than 3 standard deviations below the group mean, leaving a sample of 19 individuals.

**Fig. 5:**
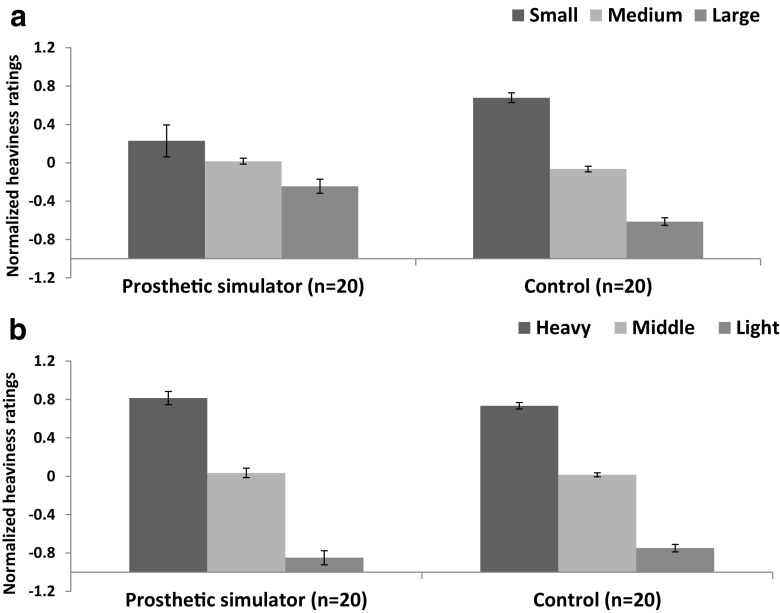
Heaviness ratings, normalized to a Z distribution within subject, as a function of (**a**) relative object size and (**b**) relative object weight. Error bars show standard error of the mean

Using the same analytic strategy as Experiment 1, we once again observed significant main effects of size, *F*(1.7, 60.2) = 163.4, *p* < .001, ω^2^ = .69 (see Fig. 5a), and weight, *F*(2, 74) = 890.6, *p* < .001, ω^2^ = .96 (see Fig. 5b), indicating that our samples experienced illusory and real weight differences. Although there was no main effect of group (*p* = .71), there was a significant interaction between size and weight, *F*(2.7, 100.4) = 5.6, *p* < .005, ω^2^ = .09, as well as a significant Size × Weight × Group interaction, *F*(2.7, 100.4) = 7.9, *p* < .001, ω^2^ = .14. We observed a significant Size × Group interaction, *F*(1.6, 60.2) = 36.2, *p* < .001, ω^2^ = .15, suggesting that, like Experiment 1, there were differences in the magnitude of the SWI experienced by our different groups. Although, the Weight × Group interaction did not reach significance (*p* = .055), it is worth acknowledging the marginal nature of the effect.

**Fig. 6:**
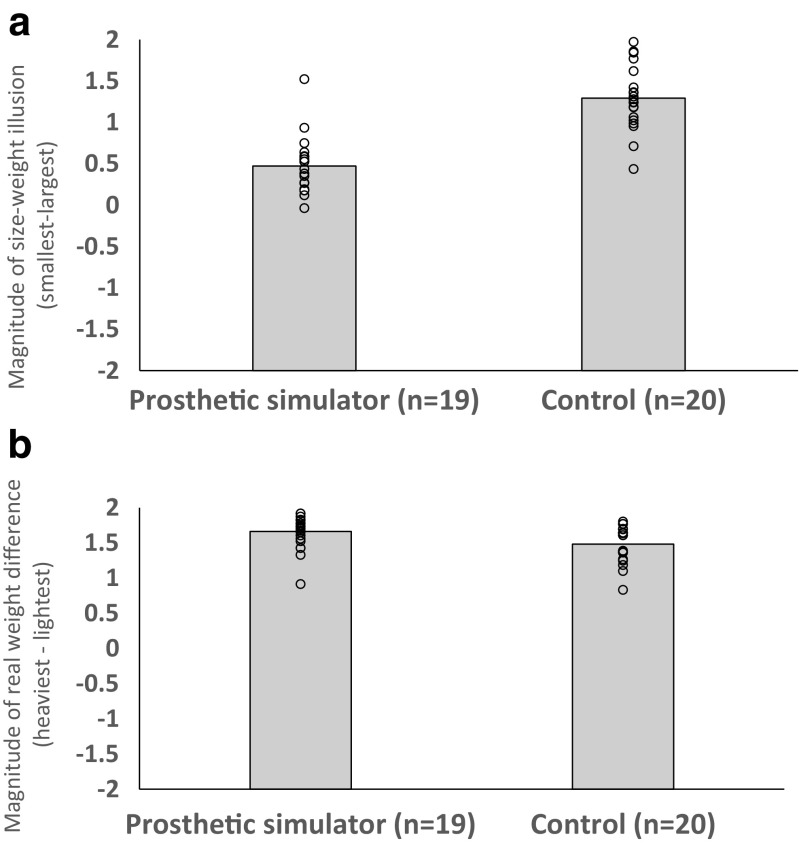
**a** Magnitude of the size–weight illusion experienced by both groups (calculated by subtracting the average ratings given to the largest objects from the average rating given to the smallest objects). **b** Magnitude of the real weight difference experienced by both groups (calculated by subtracting the average rating given to the lightest objects from the average rating given to the heaviest objects). Positive numbers indicate an effect in the expected direction (i.e., experiencing heavy objects as feeling heavier than lighter objects and experiencing smaller objects as feeling heavier than larger objects). Circles show individuals’ difference scores for these metrics

We next calculated a series of difference scores to better visualise how the magnitude of the perceptions of (1) the SWI and (2) real weight differences varied between our groups. Independent-samples *t* tests confirmed that the prosthetic simulator group experienced a smaller SWI than controls, *t*(37) = 6.9, *p* < .001, *d* = 2.21 (see Fig. 6a). In contrast to Experiment 1, the prosthetic simulator group experienced a slightly larger weight difference than the control group, *t*(37) = 2.2, *p* = .03, *d* = 0.71 (see Fig. 6b). Finally, examining these metrics in a 2 (metric) × 2 (group) mixed-factorial ANOVA confirmed that, once again, the dissociation between real and illusory weight differences experienced by these groups was significant (i.e., there was a significant interaction between metric and group, *F*(1, 37) = 31.3, *p* < .001, ω^2^ = .44.

## Comparison of Experiment 1 and Experiment 2

The findings from both experiments suggest that using a prosthetic device reduces participant’s experience the SWI, independent from their experience of real weight differences. Although these findings are in concordance with one another, directly comparing the amputee and intact prosthetic users might shed light on whether this reduced SWI varied as a function of time using/experience with a prosthetic device. To this end, we directly compared the prosthetic-using groups from Experiment 1 and Experiment 2 with the same 3 (size) × 3 (weight) × 2 (group) mixed-factorial ANOVA as above. Of most relevance to the question of interest, we observed no Size × Group interaction, *F*(2, 52) = 0.03, *p* = .97, ω^2^ = 0.0, and no Weight × Group interaction, *F*(2, 52) = 2.61, *p* = .083, ω^2^ = 0.004, suggesting that there were no differences in how long-term and short-term prosthetic users experienced the SWI or a real mass difference.

## Discussion

Here, in order to determine how hedonic perception is affected by incorporating a upper-limb prosthesis into one’s body schema, we examined how the use of a prosthetic hand affected the perception of real and illusory weight differences. We found that although prosthetic users experienced a real weight difference in a way which was indistinguishable from the control participants, their experience of the SWI was markedly reduced compared to nonamputees. This novel dissociation between the perception of real and illusory weight differences was also observed in a separate group of intact individuals who lifted the objects using a prosthetic simulator.

The observation that long-term prosthetic users experience real weight differences in an equivalent fashion to individuals using their anatomical hand is in line with our predictions and earlier work showing that a single prosthetic user has similar levels of perceptual sensitivity to controls (Wallace et al., 2002). By contrast, users of a prosthetic-simulator device in our study experienced a 200-g weight difference as more intense than individuals lifting with their anatomical hand. This latter finding is particularly surprising as the prosthetic-simulator group were using a far heavier ‘lifting effector’ (comprising the mass of the myoelectric simulator in addition to the mass of their anatomical hand) to experience the weight of the stimuli than the control group, which should have made the 200-g weight difference feel proportionally smaller than it did for the controls. This tendency to overemphasise real differences in object mass may be a consequence of how novice prosthetic users interpret the novel forces and torques induced on the anatomical contact points with the simulator by the differences in object mass as they were lifted, and points toward a mechanism by which embodiment might be functionally obtained. Regardless of the specific mechanism underpinning this effect, the perceptual experience of objects feeling subjectively heavy might impact subsequent motor performance in the context of passing items from the prosthetic to the anatomical hand (Buckingham et al., 2012; Green, Grierson, Dubrowski, & Carnahan, 2010) and be a novel contributing factor towards the relatively high rejection rate of upper-limb prosthetics (Biddiss & Chau, 2007).

Against our predictions, however, our upper-limb amputees showed a markedly smaller SWI than their control group. Indeed, by contrast to the perception of real weight difference, the way in which prosthetic use impacted the perceptual SWI were very consistent across both experiments, with both the prosthetic-using amputees and the nonamputee users of a myoelectric prosthetic simulator experiencing a markedly smaller SWI than individuals lifting with their anatomical hands. This difference is unlikely to have been caused by differences in the age of participants in the prosthetic and control groups, as several studies have shown that the SWI is no smaller in older individuals than in younger individuals (Buckingham, Reid, & Potter, 2017; Trewartha, Garcia, Wolpert, & Flanagan, 2014). When examined in the context of the growing body of work suggesting that perceptual experience is scaled relative to relevant body properties (Haggard & Jundi, 2009; Linkenauger et al., 2013; Linkenauger et al., 2011), it is certainly feasible that the reduced SWI in individuals with anorexia nervosa reflects an impairment in the way body representation drives our perception of the world. Given that similar mechanisms have been proposed for amputation, and in particular phantom pain (Foell, Bekrater-Bodmann, Diers, & Flor, 2014; Ramachandran, 1998), a similar disruption in body-based scaling could underpin the reduced SWI in our prosthetic-using population. We aim to follow up this work with a detailed investigation of how individuals level of comfort and familiarity with their prosthetic limbs, as proxies for embodiment, might relate to this counterintuitive perceptual ‘improvement’ (Marasco, Kim, Colgate, Peshkin, & Kuiken, 2011; Murray, 2008).

The current work also adds to our fundamental understanding of the perceptual SWI, confirming neuroimaging work suggesting that our experience of the SWI is fundamentally distinct from our perceptions of real weight differences (Chouinard, Large, Chang, & Goodale, 2009) and calling into question simple models of this perceptual effect. The prevailing view of the SWI is that it highlights the unique way in which our prior expectations (in this case, that small objects will weigh less than large objects) influence our experience of object weight (Buckingham, 2014; Flanagan et al., 2008)—a view which is consistent with the few special populations who experience a reduced SWI. Beyond the work outlined above on anorexia nervosa, the other notable special population which has been shown to experience a reduced SWI is patients with schizophrenia (Williams, Ramachandran, Hubbard, Braff, & Light, 2010)—an effect the authors interpret as being due to this group’s well-established deficit in forward models (Blakemore, Smith, Steel, Johnstone, & Frith, 2000; Shergill, Samson, Bays, Frith, & Wolpert, 2005). Similar conclusions were also drawn based on the findings of our recent investigation of IW, an individual with peripheral deafferentation (Buckingham, Michelakakis, & Cole, 2016), who reported no SWI or predictive lifting behaviour despite an unimpaired experience of a real weight difference (Miall, Ingram, Cole, & Gauthier, 2000), presumably reflecting a failure to incorporate prior expectations into his perceptual and motor plans. Alternatively, given that prosthetic hands have no fingertip afferents, it may well be that there is an as-yet-undefined specific role for cutaneous feedback in inducing the SWI, independent from the perception of real weight differences. When examined in this light, the reduced SWI for both of our prosthetic groups could highlight the effector-specific nature of this illusion, with the forearm (where the prosthetic is attached) as the main point of information about mass somehow dulling this perceptual effect compared to equivalent lifts with the hand. Indeed, this proposal would be consistent with earlier work showing that the SWI is experienced more robustly in the nondominant hand than the dominant hand, presumably reflecting the dominant hand’s increased perceptual sensitivity over its counterpart (Buckingham et al., 2012), and might be a more parsimonious explanation for why a reduction in peripheral input seems to selectively target the experience of the SWI (Buckingham et al., 2016). Further work involving targeted impairment of cutaneous feedback and longitudinal observations of how perception evolves when becoming expert with a hand-like tool is necessary to confirm how these cues contribute to the SWI.

Finally, one novel aspect of our study that we examined our perceptual tasks in intact individuals using a myoelectric prosthetic simulator, as well as long-term amputee prosthetic users. When we directly compared the perceptual experiences of these groups, they were indistinguishable. Our findings go some way to validating the study of intact individuals using a myoelectric prosthetic simulator as an effective surrogate to examine certain visuomotor behaviours in this traditionally difficult-to-recruit population (Parr, Vine, Harrison, & Wood, 2017). Furthermore, comparing amputee and nonamputee populations with these methods might shed light on the time course of changes in body representation which occur as the prosthesis incorporated into the body schema.

## Footnotes

1 This interaction remained significant even after removing two prosthetic users who experienced, unusually, an inverted SWI, *F*(1.5, 37.9) = 3.96, *p* = .038.
